# Enhancing the work engagement of frontline nurses during the COVID-19 pandemic: the mediating role of affective commitment and perceived organizational support

**DOI:** 10.1186/s12912-023-01623-z

**Published:** 2023-12-01

**Authors:** Yawei Shan, Xuemei Zhou, Zhiyi Zhang, Weijia Chen, Ru Chen

**Affiliations:** 1 School of Nursing, Shanghai University of Traditional Chinese Medicine, No. 1200, Cailun Road, Shanghai, 201203 China; 2 Department of Nursing, Shanghai Hongkou Mental Health Centre, Shanghai, China; 3 Department of Nursing, Changshu No. 2, People’s Hospital, Jiangsu, China; 4 Department of Nursing, Shanghai Guanghua Hospital of Integrative Medicine, Shanghai University of Traditional Chinese Medicine, Shanghai, China

**Keywords:** Affective commitment, COVID-19, Perceived organizational support, Work engagement, Nurses

## Abstract

**Background:**

Overload and anxiety were common phenomena among frontline nurses during the pandemic. Understanding the potential pathway for fostering engagement in high-stress working conditions can provide evidence of targeted intervention to facilitate nurses’ well-being and safety practices. This study aims to investigate the level of nurses’ work engagement during nucleic acid collection tasks in the COVID-19 pandemic and identify its potential antecedents.

**Methods:**

A cross-sectional design was adopted. A sample of 824 nurses who engaged in nucleic acid collection tasks completed an online self-report questionnaire between 1 March and 31 May 2022. Descriptive and path analyses were utilized to analyse the interrelationships among anxiety, perceived workload, affective commitment, perceived organizational support and work engagement. This study was conducted and reported under the guidelines for Strengthening the Reporting of Observational Studies in Epidemiology.

**Results:**

The results showed that frontline nurses engaged in such tasks reported high levels of anxiety and task load and low levels of work engagement. Path analysis identified anxiety symptoms, perceived workload, perceived organizational support, and affective commitment as associated with work engagement, and among these factors, perceived organizational support and affective commitment played key roles in mediating the relationship of anxiety, workload and work engagement in high-stress working conditions.

**Conclusions:**

Affective commitment and perceived organizational support were associated with frontline nurses’ level of work engagement during the COVID-19 pandemic; these two variables might explain how engagement is generated in high-anxiety and high-workload situations. When healthcare organizations give more attention to frontline nurses’ physical and psychological conditions and are able to innovatively motivate affective commitment and facilitate organizational support, nurses’ work engagement in high-level tasks may increase, thus enhancing work safety and personal well-being.

**Supplementary Information:**

The online version contains supplementary material available at 10.1186/s12912-023-01623-z.

## Background

The COVID-19 pandemic was considered a major public health emergency and constituted a massive health and socioeconomic challenge worldwide [1]. Nurses played a vital role in this pandemic. Frontline nurses struggled with the impact of COVID-19 on multiple fronts that represent areas of ethical concern, such as heavy workloads, threats to safety, work-family conflict, scarce resource allocation and complex relationships with public citizens [2]. Studies have reported that frontline nurses are at risk of suffering from psychological disorders [3], such as insomnia, anxiety, depression and posttraumatic stress disorder [4]. This phenomenon may trigger situations that present negative behavioural consequences for nurses, such as unsafe practices, virus infection and a higher rate of job turnover [5].

During the outbreak of COVID-19 caused by the SARS-CoV-2 Omicron BA.2 sublineage from 1 March to 31 May, 2022 in Shanghai, China, nucleic acid collection (nasopharyngeal swab and midturbinate swab) was considered one of the highest-intensity tasks [6]. During this period, more than 56,000 infections were confirmed in Shanghai through frequent screening [7]. As reported on the first day of nucleic acid collection and testing in Shanghai, approximately 25,000,000 samples were collected by 40,000 healthcare professionals (approximately 80% were nurses) [7]. With the ever-increasing number of infections, nurses undertaking nucleic acid collection suffered both psychological and physical pressure due to high-intensity tasks, a high risk of infection [4], long working hours, social isolation, disturbed sleep, restrictive personal protective equipment (protective suit, face shield and N95 mask) worn in increasing outdoor temperatures, a lack of sterilization facilities for outdoor work conditions, and a lack of fixed workplace and time [8]. These conditions differed from other nursing tasks or even the same task conducted in the context of a hospital or quarantine sites. Drawing insights from previous studies that have provided evidence of the working status and its factors associated with inpatient nurses [9], emergency nurses [10] and nurse managers [11], there is a scarcity of research focusing on nurses engaged in nucleic acid collection. The level of work engagement of the nurses involved in this kind of task might be greatly affected and warrants investigation.

Work engagement is an optimistic, satisfying work-related state of mind consisting of cognitive, physiological and emotional engagement, which is characterized by vigour, devotion and commitments in certain tasks [12]. Studies have indicated that a high level of work engagement could promote task performance and job satisfaction [9, 12]. During the COVID-19 pandemic, nurses’ work engagement in patient care was greatly influenced by several factors and resulted in negative outcomes, including a high level of intention to leave and a high rate of turnover [13]. However, little is known about the status and potential antecedents of work engagement among nurses undertaking nucleic acid collection tasks. As the pandemic situation was described as volatile, uncertain, complex, and ambiguous [14], understanding the level of nurses’ work engagement in nucleic acid collection tasks and how such engagement was fostered in high-stress working situations can provide evidence for targeted interventions to facilitate nurses’ well-being and safety practices and retain qualified nurses. In addition, identifying the potential facilitators and barriers to nurses’ work engagement could contribute to the literature on applied psychology during large-scale public health events.

### Relationships among anxiety, perceived workload and work engagement

The current literature on work engagement or burnout mainly follows the theory of job demands and resources, which focuses on job demands and internal and environmental resources [15]. Several studies have investigated the predictors of employees’ work engagement during the COVID-19 pandemic [16, 17], focusing on either work overload or negative psychological conditions that impair individuals’ repertoires of resources, thus reducing their work engagement [18]. However, Chang et al. [19] found that work overload does not determine negative work engagement in medical crises. Therefore, findings on the relationship between workload and work engagement are inconsistent. Workload is the number of tasks that reflects the effort required of nurses to meet physical, temporal and environmental demands. Work overload is a common phenomenon in COVID-19 pandemic healthcare tasks [20], while nurses’ workload in nucleic acid collection tasks has not been assessed in any recent studies. Although workload accelerates with job intensity and working hours, individuals with different psychological conditions undertaking the same task in the same organization may perceive their workloads differently. Therefore, workload should be measured in subjective, rather than objective, terms. However, the investigation of exact measures of subjectively perceived workload could provide basic characteristics of the task that permit comparisons and provide a reference for relevant studies. Regarding negative psychological reactions, the risk and fear of contagion resulting from a massive number of confirmed cases and a high-risk environment may increase nurses’ anxiety level [21], thus influencing the level of perceived workload. Consequently, a decline in work engagement might occur in such a physically and emotionally challenging context [22]. As such, we proposed the following hypotheses:

Hypothesis 1: Anxiety is negatively associated with nurses’ work engagement.

Hypothesis 2: Perceived workload is positively associated with nurses’ work engagement.

Hypothesis 3: The level of anxiety is positively associated with nurses’ perceived workload.

### Mediating effect of perceived organizational support

In the context of job demands and resources theory [22], perceived organizational support acts as an environmental resource that reduces the effect of nurses’ anxiety on work engagement. Concerning the required working environment for nurses, involving isolation and social distancing to limit virus spread, environmental resources, especially perceived organizational support, might substantially and positively influence work engagement in this context [23]. Perceived organizational support is defined as an employee’s perceptions that his or her effort and contribution are appreciated by his or her organization [24]. Perceived organizational support reflects employee judgements about the organizational environment, including fairness, human resources, and leadership, which are linked to organizational commitment and engagement [25]. Some studies have broadly elucidated the mediating role of organizational support in the effect of anxiety on work engagement [26]. As reported in a study among nurses in Turkey, organizational support could mediate the effect between workload and affective commitment [20]. In our investigation, which was conducted during the highest peak of the outbreak, the dramatic burden of workload could not be avoided due to limited professional healthcare resources. Therefore, we assert the following hypothesis:

Hypothesis 4: Perceived organizational support mediates only the relationship between anxiety symptoms and work engagement.

### Mediating effect of affective commitment

Affective commitment is a positive emotion reflecting the degree of an employee’s identification, involvement and affective attachment to the organization [27]. According to the three domains of work engagement, affective commitment reflects emotional engagement [17]. Therefore, a higher level of affective commitment could predict positive organizational behaviour, such as working voluntarily and with passion [28]. According to the unique sensory processing pattern among psychological disorder groups, negative emotion can result in compromised affective commitment [29]. A recent investigation of the working status of psychiatrists during the COVID-19 pandemic reported a relationship between negative emotion and affective commitment [30]. Moreover, as a high workload could result in insufficient or a lack of personal resources to meet job-related commitments and requirements under the strength model [31], employees might lose confidence and trust in their organizations; thus, positive emotional bonds between individuals and organizations might be eroded [20]. Drawing upon theories and previous studies, anxiety and perceived workload are presumed to be negatively related to affective commitment. In addition, previous studies have indicated a relationship between perceived organizational support and affective commitment based on social identity and social exchange theory [32], and investigations of workplace support during the COVID-19 pandemic have found that perceived organizational support is associated with more positive practice behaviours, which are mediated by employees’ affective commitment [25]. Therefore, we assert the following hypotheses:

Hypothesis 5: Affective commitment plays a mediating role in the relationship between anxiety and work engagement.

Hypothesis 6: Affective commitment plays a mediating role in the relationship between perceived workload and work engagement.

Hypothesis 7: Affective commitment plays a mediating role in the relationship between organizational support and work engagement.

The aim of this study was to investigate the level of nurses’ work engagement in nucleic acid collection tasks, identify the antecedents of work engagement and explore how such work engagement is fostered in high-stress working situations according to the above hypotheses.

## Methods

### Design, participants and setting

A cross-sectional self-report study was conducted during the largest lockdown in Shanghai, China. Frontline nurses undertaking nucleic acid collection tasks, including nasopharyngeal swabs and midturbinate swabs, were recruited online from 1 March to 31 May 2022. All participants were registered nurses and employed full time in various departments of urban hospitals at different levels. Participants were from 13 different districts of Shanghai city, which consists of 16 districts. Nurses volunteered to conduct the task and were formally trained to perform nucleic acid tests. According to Cohen’s sample size recommendation [33] and using G*Power [34] and the 20 times rule of thumb, a minimum of 260 participants were needed for this study. The study setting was a nucleic acid collection outdoor facility where nurses undertook nucleic acid collection tasks for the residents of the city. Day shifts were generally scheduled from 8:00 A.M. to 20:00 P.M. depending on the number of residents, night shifts were from 20:00 P.M. to 0:00 A.M., and midnight shifts were from 0:00 A.M. to 8:00 A.M. in emergencies. During the task, nurses would travel to several communities to collect nucleic acid samples and wear protective equipment, including personal protective suits, face shields and N95 masks; however, personal sterilization facilities were lacking or absent.

### Data collection and measurements

Data were collected from 1 March to 31 May through an online questionnaire platform called *Wenjuanxing* (www.wjx.cn), on which only a fully completed questionnaire could be uploaded. Initial permission was sought and obtained from various department heads and hospital administrators before the release of the recruiting information and questionnaire, and participation was entirely voluntary with online consent. Frontline nurses reported their data according to their condition in the last 2 weeks because the mean interval of nucleic acid collection tasks for individuals is 2 weeks. Data collection was discontinued when the data were not uploaded within 7 days.

### Self-designed questionnaire for sociodemographic characteristics

The self-designed questionnaire included sociodemographic characteristics, including sex, age, marital status, number of children, years of clinical practice, educational level, professional title of the nurse, original practice department and hospital level.

### Measurement of subjective workload

The subjective workload of nurses engaged in nucleic acid collection tasks was assessed using a self-designed questionnaire on workload in the last two weeks that included (1) working days for tasks per week, (2) samples taken per day, (3) hours of sleep per day, (4) days off per week and (5) number of midnight tasks per week.

### Measurement of perceived workload

Perceived workload was evaluated by the Chinese version of the National Aeronautics and Space Administration Task Load Index (NASA-TLX) [35], which comprises 6 subscales (or items) regarding different workload aspects (mental demands, physical demands, temporal demands, performance, effort, and frustration). The Chinese version was translated by Liang L. et al. with subscale scores ranging from 0 to 100. The nurse gives a numerical score on each of the six visual analogue subscales that best matches their experience during the task. The Cronbach’s ɑ of the Chinese version was 0.707 [36], and the Cronbach’s ɑ in this study was 0.823. In this study, we used the total mean score of the subscales of mental demands, physical demands, and temporal demands to reflect the perceived workload.

### Measurement of anxiety

Anxiety was measured by the self-rating anxiety scale with a total of 20 items that evaluated the frequency of symptoms, including mental affective symptoms, somatic disorders, psychomotor disorders and depressive psychological disorders [37]. The items were rated on a 4-point scale, where 1 was ‘no or limited time’ and 4 was ‘most or all the time’, with a higher score indicating more severe anxiety. The Cronbach’s α of the self-rating anxiety scale was 0.777 among nurses during the COVID-19 pandemic [38] and was 0.806 in this study, indicating good reliability and validity. In this study, we used the total sum score of items to reflect the level of anxiety of nurses.

### Measurement of perceived organizational support

Perceived organizational support was measured by the brief Chinese version of the perceived organizational support scale [39], which contains 8 items, including the aspects of instrumental support, emotional support and institutional protection. The items were rated on a 5-point Likert scale, where 1 was ‘strongly disagree’ and 5 was ‘strongly agree’, and higher scores indicated better organizational support. The Cronbach’s α of this scale was 0.830 in a relevant study [40] and was 0.862 in this study. In this study, we used the total sum of scored items to reflect the level of perceived organizational support of nurses.

### Measurement of affective commitment

Affective commitment was measured by the affective subscale from the organizational commitment questionnaire (affective commitment, normative commitment and sustained commitment) developed by Meyer et al. [41]. The affective commitment subscale consists of 6 items rated on a 5-point Likert scale, where 1 was ‘strongly disagree’ and 5 was ‘strongly agree’, with higher scores indicating higher affective commitment. The Chinese version of the affective commitment subscale was verified during the COVID-19 pandemic with a Cronbach’s α of 0.904 [30]. In this study, the Cronbach’s α of this scale was 0.912, and we used the total sum of scored items to reflect the level of affective commitment of nurses.

### Measurement of work engagement

Work engagement was measured using the brief Chinese version of the work engagement scale developed by Schaufeli et al. [42] and translated by Wang et al. [43]. The original instrument consists of three dimensions (vigour, dedication and absorption) with 18 items. In this study, the brief Chinese version of the work engagement scale was one dimensional and consisted of 9 items rated on a 5-point Likert scale, on which 1 was ‘strongly disagree’ and 5 was ‘strongly agree’, with higher scores indicating higher work engagement. The Cronbach’s α of the brief Chinese version was 0.908 [44]. In this study, we used the total sum of scored items to reflect the level of work engagement of nurses, and the Cronbach’s α of this scale was 0.920.

### Validity and reliability/rigour

Chinese versions of measurement tools with good validity and reliability were selected, and the psychometric properties of the tools are described above. The Cronbach’s α of each scale is higher than 0.70 in this study, suggesting a reliably high internal consistency. Permission for use was acquired before the investigation. This study was conducted and reported under the Strengthening the Reporting of Observational Studies in Epidemiology (STROBE) guidelines [45].

### Ethical considerations

Guided by the 2000 Declaration of Helsinki for ethical standards, the protocol was approved by the Committee on the Ethics of Medical Research of ** centre (No. 2022-Q**1). Informed consent was provided by participants prior to their participation. The survey was anonymous, and the confidentiality of the information was assured.

### Statistical analysis

Statistical analysis was carried out with SPSS 21.0 (IBM Corp), and *p* < .05 (two-tailed) was considered significant. Descriptive statistics, including the number (n), percentage (%), mean (M) and standard deviation (SD), were used to analyse the sociodemographic and occupational characteristics, subjective workload, perceived workload, anxiety, perceived organizational support, affective commitment, and work engagement. Normality and equal variance for all the variables were checked using Shapiro–Wilk and Bartlett’s tests. Pearson’s correlation was applied to evaluate correlations between variables according to the hypothetical model. Path analysis was performed with the maximum-likelihood method implemented in SPSS Amos v23.0 (IBM Corp) to examine the mediating role. The basic assumptions for modelling included linearity, causal closure, unitary variables, the endogenous variables being continuous and normally distributed and covariances among the disturbance terms being zero. Fit indices were examined to determine the appropriate model, including χ^2^, root mean square error of approximation (RMSEA), goodness-of-fit index (GFI), adjusted GFI (AGFI), comparative fit index (CFI), Tucker‒Lewis index (TLI), normed fit index (NFI) and incremental fit index (IFI). Squared multiple correlations (*R*^2^) were calculated to reflect the proportion of variance in work engagement that was accounted for in the full model. The path coefficient estimated the intensity between two study variables and was analysed using a standardized regression coefficient (β weight). Bootstrap resampling with 10,000 samples was performed.

## Results

### Participant characteristics and descriptive statistics of the variables

A total of 940 nurses participated, among whom 98 nurses were excluded, as they reported that they did not undertake nucleic acid collection tasks in the subjective workload questionnaire. The number of valid responses was 842 (89.6%) without any missing data. The sociodemographic characteristics of the participants are shown in Table 1. Table 2 provides the subjective workload of participants over two weeks, and 702 (83.4%) nurses performed both nucleic acid collection tasks and their original work. Table 3 provides the means, SDs and Pearson’s correlation coefficients of all variables.

**Table 1:** Sociodemographic and occupational characteristics of nurses (n = 842)

Characteristic		Number	Percentage (%)
Overall		824	100.0
Sex	Men	26	3.1
	Women	816	96.9
Age, y	≤ 25	185	22.0
	25 ~ 35	403	47.9
	35 ~ 45	186	22.1
	> 45	68	8.1
Marital status	Unmarried	541	64.3
	Married	301	35.8
No. of children	≥ 1	488	58.0
	0.00	354	42.0
Years of clinical practice, y	≤ 5	250	29.7
	6 ~ 10	196	23.3
	11 ~ 15	169	20.1
	16–20	107	12.7
	> 20	120	14.3
Educational level	Associate’s degree	285	33.9
	≥Bachelor’s degree	557	66.2
Hospital level	Primary	116	13.8
	Secondary	376	44.7
	Tertiary	350	41.6
Practice department	Emergency and intensive care	54	6.41
	Operating room	56	6.7
	Outpatient department	244	29.0
	Medical department	289	34.3
	Surgical department	199	23.6
Professional title	Junior	493	58.6
	Intermediate	313	37.2
	Senior	36	4.3

**Table 2:** Subjective workload of nucleic acid collection tasks over two weeks (n = 842)

Workload	M ± SD	Range (P_25_, P_75_)
Days for tasks per week	5.40 ± 4.51	0.50, 14.00(1.50, 8.00)
No. of samples taken per day	543.80 ± 703.69	120.00, 1000.00(150.00, 600.00)
Days off per week	0.27 ± 0.29	0.00, 1.00(0.00, 1.00)
Hours of sleep per day	6.31 ± 1.20	0.50, 10.00(0.50, 10.00)
Number of midnight tasks per week	1.62 ± 2.63	0.00, 14.00(0.00, 14.00)

Note: M = mean; SD = standard deviation; P = percentile

**Table 3:** Descriptive statistics and correlations among perceived workload, anxiety, organizational support, affective commitment and work engagement (n = 842)

Variables		M ± SD	Range	1	2	3	4	5
1	Perceived workload	69.23 ± 16.35	25.00, 100.00	1				
2	Anxiety	42.27 ± 10.97	20.00, 80.00	0.03^**^	1			
3	Organizational support	26.10 ± 4.49	18.00, 40.00	− 0.07^*^	− 0.25^**^	1		
4	Affective commitment	19.44 ± 4.42	12.00, 25.00	− 0.04	− 0.43^**^	0.53^**^	1	
5	Work engagement	32.13 ± 7.55	9.00, 45.00	− 0.03	− 0.42^**^	0.51^**^	0.73^**^	1

Note: M = mean; SD = standard deviation; ^*^p < .05, and ^**^p < .01

### Path analysis of variables

According to the path analysis, the best-fitting model included the variables of perceived workload, anxiety, perceived organizational support, affective commitment, and work engagement. The fit indices of the full model were χ^2^ = 3.638, *df* = 2, *p* = .162, RMSEA = 0.031, GFI = 0.998, AGFI = 0.987, CFI = 0.999, TLI = 0.994, NFI = 0.997, and IFI = 0.999, indicating that the model fit the data well. The *R*^2^ value, reflecting the proportion of variance in work engagement accounted for by the full model, is 0.611.

As recommended by Preacher and Hayes [46], a three-step approach was employed to test the hypotheses. (1) The overall effect of anxiety and perceived workload on work engagement was analysed without an intermediary variable (Model 1 in Appendix Tables 1 and 2). The results showed that anxiety symptoms (standardized total effect: β = − 0.456, SE = 0.031, 95% CI= [-0.516, − 0.394], *p* < .001) and perceived workload (standardized total effect: β = 0.120, SE = 0.035, 95% CI= [0.050, 0.186], *p* = 002) were associated with nurses’ work engagement. In addition, symptoms of anxiety had significant positive correlations with nurses’ perceived workload (standardized total effect: β = 0.327, SE = 0.030, 95% CI= [0.265, 0.385], *p* < .001). Therefore, Hypotheses 1 to 3 were supported.

**Fig. 1:**
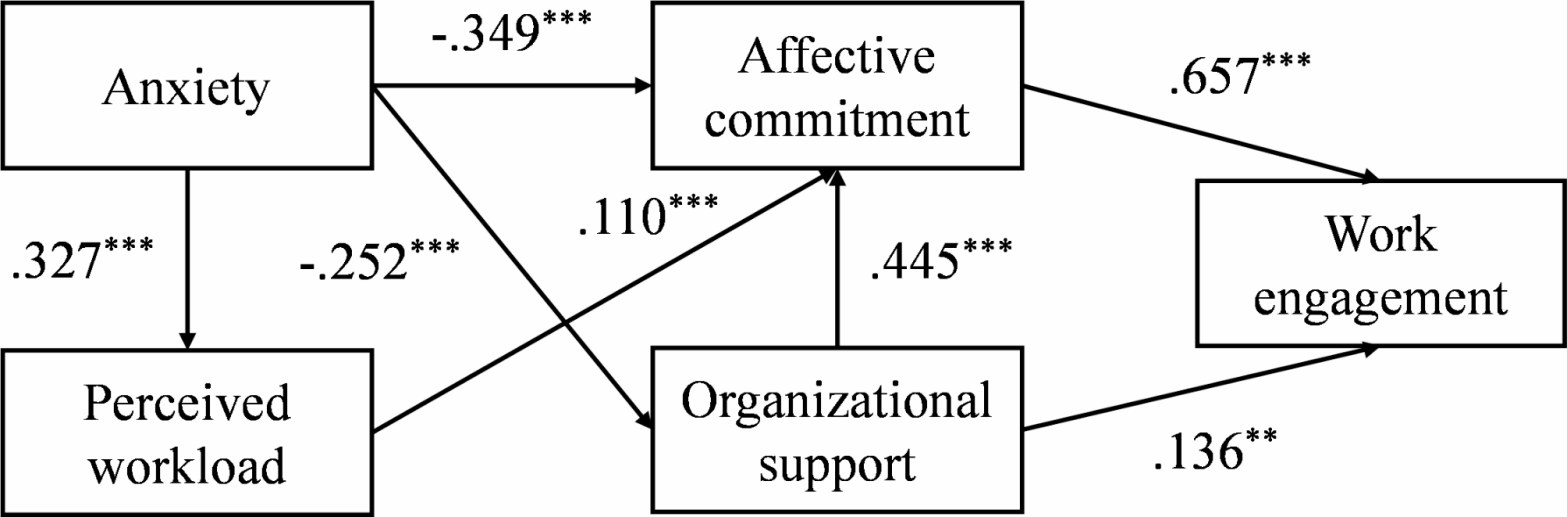
Structural model of the variables *Note:* *** *p* < .001; ** *p* < .01

(2) The relationships among anxiety, perceived workload, affective commitment, and job engagement were examined (Model 2 in Appendix Tables 1 and 2). The results showed that affective commitment played a mediating role in the relationship between anxiety and work engagement (standardized indirect effect: β = − 0.328, SE = 0.028, 95% CI= [-0.382, − 0.272], *p* < .001) and in the relationship between perceived workload and work engagement (standardized indirect effect: β = 0.090, SE = 0.025, 95% CI= [0.041, 0.139], *p* < .001). Therefore, Hypotheses 5 and 6 were supported. The same steps were employed to test the relationships among anxiety, perceived organizational support and work engagement (Model 3 in Appendix Tables 1 and 2). The results showed that perceived organizational support mediated the relationship between anxiety symptoms and work engagement (standardized indirect effect: β = − 0.071, SE = 0.019, 95% CI= [-0.109, − 0.033], *p* < .001). Therefore, Hypothesis 4 was supported.

(3) The full model was tested with all the variables (Full Model in Table 4 and Appendix Tables 1 and 2). The results of the full model showed that the standardized indirect effect of organizational support on work engagement via affective commitment was 0.293 (SE = 0.023, 95% CI= [0.247, 0.337], *p* < .001). Therefore, Hypothesis 7 was verified. The detailed pathway and standardized direct effect estimates are shown in Fig. 1, and the full model is shown in Table 4.

**Table 4:** Results of the standardized total effect, direct effect and indirect effect estimates of variables on work engagement in the full model

Significant path	Effect	SE	LLCI	ULCI	*p*
Total effect: Anxiety --> Work engagement	-0.314	0.027	-0.366	-0.262	< 0.001
Total effect: Perceived workload --> Work engagement	0.072	0.02	0.034	0.111	< 0.001
Total effect: Organizational support --> Work engagement	0.429	0.033	0.358	0.489	< 0.001
Direct effect: Anxiety --> Perceived workload	0.327	0.03	0.265	0.385	< 0.001
Direct effect: Anxiety --> Organizational support	-0.252	0.032	-0.311	-0.186	< 0.001
Direct effect: Anxiety --> Affect commitment	-0.349	0.033	-0.411	-0.282	< 0.001
Direct effect: Perceived workload --> Affect commitment	0.110	0.029	0.052	0.167	< 0.001
Direct effect: Organizational support --> Affect commitment	0.445	0.029	0.384	0.5	< 0.001
Direct effect: Organizational support --> Work engagement	0.136	0.032	0.066	0.195	0.001
Direct effect: Affect commitment --> Work engagement	0.657	0.026	0.605	0.705	< 0.001
Indirect effect: Anxiety --> Work engagement	-0.314	0.027	-0.366	-0.262	< 0.001
Indirect effect: Perceived workload --> Work engagement	0.072	0.02	0.034	0.111	< 0.001
Indirect effect: Organizational support --> Work engagement	0.293	0.023	0.247	0.337	< 0.001

## Discussion

### Key findings

This study was conducted during the largest lockdown of a metropolitan city during the peak of the COVID-19 pandemic. To our knowledge, this is the first study to investigate the level of nurses’ work engagement in nucleic acid collection tasks and explore the relationships among affective commitment, perceived organizational support and work engagement in high-anxiety and work overload healthcare contexts. According to the investigation of subjective workload, nurses involved in nucleic acid collection tasks during this peak outbreak period had a heavy workload, a high incidence of anxiety symptoms and a low level of work engagement. Path analysis identified that perceived workload, symptoms of anxiety, perceived organizational support, and affective commitment were associated with work engagement, among which perceived organizational support and affective commitment played key roles in mediating the relationship between anxiety, work overload and work engagement in high-stress working conditions. As no single study has explored the relationship between anxiety, workload and work engagement using perceived organizational support and affective commitment as mediators, the findings of our study could provide a theoretical reference for applied psychology on large-scale public health events and contribute critical insights for both nursing researchers and managers.

### Interpreting the findings

#### Lower work engagement was reported by nurses involved in nucleic acid collection tasks

As studies have established a strong link between work engagement and sense of duty, work performance and unsafe working behaviours [12], efforts to reduce negative psychological and performance outcomes are continuously being studied and implemented [9]. The results of this study showed that nurses engaged in this kind of arduous task had a lower level of work engagement than those in a previous study who performed other tasks [4]. The evident decline in work engagement in nucleic acid collection might be one of the reasons for the high rate of infection among those nurses [4]. Particularly, during the citywide lockdowns in this crisis, nurses had to travel to several communities to collect nucleic acid samples, while the necessary personal sterilization and bathing services were absent due to limited facilities [47]. Enhancing engagement in tasks and caution in personal protection could prevent infection and facilitate task performance. Therefore, our study revealed Chinese nurses’ devotion to pandemic prevention and control and provided clues for enhancing the positive factors of work engagement by identifying key related factors.

#### Anxiety and work overload among nurses were associated with work engagement

In this study, the total mean level of anxiety scores (42.27 ± 10.97) for nurses engaged in nucleic acid collection tasks was significantly higher than that for frontline nurses involved in fighting COVID-19 during the first outbreak in Wuhan in 2020 (32.19 ± 7.56) and was much higher than the national standard score (29.78 ± 0.46) [38], indicating that nurses experienced evident anxiety during this epidemic outbreak. Our structural model of variables confirmed Hypothesis 1, which stated that anxiety was negatively associated with nurses’ work engagement. This finding is in line with the job demands and resources model and most of the empirical findings [4, 10]. Concerning the reason for the high level of anxiety, although COVID-19 sublineages (SARS-CoV-2 BA.2.2) have evolved towards less virulence, a higher rate of contagious and severe outcomes among unvaccinated people and older adults has been reported [7]. With ever-increasing infections, nurses’ feelings of insecurity might be one of the major causes of anxiety [16]. Moreover, since the shortage of nurses during the COVID-19 pandemic was a prominent phenomenon in healthcare [14], nurses may have suffered from overload, anxiety and low work engagement prior to nucleic acid collection tasks. In addition, the sociodemographic and occupational characteristics of the nurses in this study showed that the majority of participants were junior nurses who were single and may have lacked experience, maturity, critical thinking skills, and social support at the novice level. Regarding the pandemic context, local citizens suffered inconveniences in their daily lives due to lockdowns, and some people even developed mental health symptoms; worrying about family members’ material, physical, and psychological health might have prevented nurses from investing their full selves at work. Therefore, the negative association between anxiety and work engagement might reflect the importance of support both in infection prevention and the daily needs of nurses and their families.

The work overload of nurses undertaking nucleic acid collection tasks was a prominent situational factor that influenced nurses’ work engagement. Because of increasing test numbers and nurse shortages, 83.4% of nurses had to juggle their usual nursing care in hospitals and nucleic acid collection tasks in communities. According to the subjective workload reported by participants, the average number of samples taken per day (543.8) by one nurse indicated that they had to continuously work for more than 8 h, and considering the wearing of personal protective equipment and increasing outdoor temperatures, the tasks were both intensive and arduous. Moreover, the findings in this investigation for average days off per week (0.27), hours of sleep per day (6.31) and number of midnight tasks per week (1.62) indicated that most nurses suffered from rest and sleep deprivation and could receive emergency orders at any time, suggesting that the workload in nucleic acid collection tasks was higher than that in other tasks in pandemic control. In particular, as Hypothesis 3 stated, in accord with the empirical data, the state of anxiety was positively associated with nurses’ perception of a heavy workload, which was in line with the findings among nurses supporting the Wuhan outbreak [38]. In this study, we identified perceived workload as a potential positive mediator between anxiety and work engagement. Although a previous study emphasized that work overload is harmful, causing exhaustion and reducing work engagement [48], our result might be explained by the saying “with great power comes great responsibility”, and this result was in line with previous findings among medical residents that work overload is positively related to work engagement in medical crises [19]. Moreover, nurses may feel that their nursing oaths are tested when confronted with such a public crisis and the need for increased work engagement [3].

#### Mediating effect of affective commitment and perceived organizational support on work engagement

In this study, affective commitment and perceived organizational support were confirmed as the key mediators between anxiety, perceived workload and work engagement (Hypotheses 4 and 5), providing an important supplement to the existing research on the potential generative mechanism for work engagement in a high-stress work context. In addition, our results suggest that affective commitment and perceived organizational support might buffer the negative effect of anxiety on work engagement, which could be explained by the strength model that stressors, negative emotion and support shortages are the major reasons for ego depletion, thus causing limited resources in coping with crises [31], and affective commitment and perceived organizational support could serve as intrinsic motivational resources and environmental resources to facilitate the achievement of positive working results. A similar conclusion was also reached in a study conducted by researchers with various employee roles during the pandemic period [25]. Our study reinforces the idea of research-related job resources and contributes to management strategies by stressing the importance of the mediating function of affective commitment and perceived organizational support associated with work engagement and performance.

In our study, perceived workload in nucleic acid collection tasks was positively related to affective commitment and thus linked to work engagement. Considering the strength model, a high workload could result in insufficient job-related commitments and requirements [31], which seems to be in contrast with our study findings. However, a few studies have analysed whether employees with strong affective commitment to their career or organization have a high level of engagement, even in work overload conditions, especially during severe public health events [49], which is in line with our findings. In our study, the nurses’ mean affective commitment score was 19.44 (SD 4.42), which was higher than those of physicians or nurses in other settings [50]. However, when compared with other employees during the COVID-19 pandemic, nurses’ affective commitment to nucleic acid collection tasks was lower [49]. As a commentary from Harrison et al. states, “Creating a healthcare context that promotes clinician engagement with change remains elusive, with limited demonstrated progress”, and evidence from the healthcare context indicates that enhancing affective commitment could facilitate self-efficacy and readiness to cope with crisis [51]. Nursing managers may need to reorient the current strategies to improve affective commitment through approaches that focus on authenticity, open communication, and organizational support and that foster cultures based on shared values and priorities.

Our finding of affective commitment mediating the relationship between perceived organizational support and personal well-being (Hypotheses 6 and 7) is consistent with that in the study by Kim et al. [24] among physicians and nurses during the COVID-19 pandemic. Moreover, affective commitment contributed the largest regression weight path in the model, suggesting that the intervention of promoting affective commitment is promising for strengthening work engagement. According to the three components of affective commitment conceptualized by Mayer and Allen [41], personal dispositions, work-related factors, and organizational characteristics determine affective commitment. Therefore, organizational support might work as an important organizational characteristic enhancing nurses’ affective commitment to healthcare and work engagement [52]. Referring to a national investigation of healthcare organization actions and policies related to COVID-19 among U.S. internists [23], improving and motivating clinicians from the organizational perspective were found to be essential in facilitating their mental health and preventing burnout and turnover intention in the healthcare sector. However, the item-mean perceived organizational support score of 3.26 in our study indicated a medium level. It is unknown whether the level of perceived organizational support among nurses in this study was different from that prior to the pandemic, as no data were reported or investigated before, while compared with studies on the Wuhan pandemic, the levels of both were almost the same [53]. Moreover, among the actions or policies in response to COVID-19, leaders listening to healthcare workers’ concerns, providing adequate personal protective equipment, and avoiding warnings or sanctions for those reducing redeployment or speaking out on safety issues are principal strategies through which to improve organizational support [23].

### Implications for nursing

This study focused on nurses involved in nucleic acid collection tasks during the COVID-19 pandemic, who are the largest healthcare workforce but whose perspectives are not always fully considered. The findings of this study may have implications for nursing management in the pandemic period and even beyond the extreme case of a pandemic, such as in medical crises and other public health emergencies resulting in a heavy workload for nurses. First, it is suggested that nurses’ physical and psychological well-being in nucleic acid collection tasks, which were previously overlooked and shown to be poor, be given close attention. Mitigating the effect of those negative factors might be a potential method to reduce anxiety symptoms and thus enhance work engagement. In this context, enhancing approaches to comprehensive support for nurses could relieve their concerns. Although this study revealed a positive association between workload and work engagement, carefully scheduling tasks and ensuring adequate rest according to the results of subjective workload may optimize perceived workload. Therefore, the investigation of workload in both subjective and objective aspects could provide clues to optimizing task allocation in public health emergencies.

Moreover, our results on the mediating role of affective commitment and perceived organizational support in the relationship between anxiety and perceived workload could provide several implications. As the sense of affective commitment could bring about more behavioural motivation to involve the self in high-stress tasks, it is suggested that a harmonious organizational climate be created to exert a positive influence of affective commitment on nurses. According to the three components of affective commitment, setting up an effective communication mechanism and proposing interventions to alleviate work-family conflict are possible methods to enhance affective commitment [41]. Since affective commitment is also characterized as a personal disposition [27], nursing educators and managers are encouraged to introduce training to strengthen nurses’ altruism in healthcare. In addition, perceived organizational support has a contextual characteristic and can be changed or induced with the implementation of human resource practices. In the context of the pandemic, with tasks similar to nucleic acid collection, as psychological and physical pressure for nurses performing these tasks increases, improvements to the working environment, including the employment of negative pressure rooms and personal sterilization facilities to minimize the risk of infection, could provide major support to mitigate nurses’ anxiety symptoms; furthermore, direct instrumental support and emotional support could optimize nurses’ perceived workload. Implementing a humanistic management strategy by considering the nurse as a whole, being aware of the substantial specificity of nurses, and protecting and promoting their constitutive dignity is essential for organizational support [54].

### Limitations

This study has several limitations. (1) Convenience sampling was used to recruit participants, and the majority of the participants were female, which might limit the generalizability of our conclusions. (2) An online questionnaire platform was employed to collect data. The number of delivered questionnaires and differences between participating nurses and those who declined to participate were unclear. (3) The use of self-reported measures might have resulted in common method variance and social desirability bias. In addition, other factors affecting work engagement that were not measured were included in the proportion of variance in work engagement accounted for by the full model. (4) We found that workload was positively related to engagement, which remains controversial among different studies. (5) Causality could not be determined due to the cross-sectional design of this study.

## Conclusions

According to this survey study, frontline nurses engaged in nucleic acid collection tasks during the COVID-19 pandemic reported high levels of anxiety symptoms and task load and low levels of work engagement. Our study found that affective commitment and perceived organizational support could mediate the negative effect of the COVID-19 pandemic and play the role of key determinants of work engagement in high-stress work conditions. Healthcare organizations can innovate and inspire and motivate nurses’ affective commitment and facilitate organizational support so that they engage in high-level tasks, which could enhance work safety and personal well-being.

### Electronic supplementary material

Below is the link to the electronic supplementary material.

Supplementary Material 1

## Data Availability

The datasets used for the current study are available from the corresponding author upon request.

## Declarations

### Guidelines statement

All methods were carried out in accordance with relevant guidelines and regulations.

## Abbreviations

NASA-TLX: National Aeronautics and Space Administration Task Load Index
RMSEA: Root mean square error of approximation
GFI: Goodness-of-fit index
AGFI: Adjusted goodness-of-fit index
CFI: Comparative fit index
TLI: Tucker‒Lewis index
NFI: Normed fit index
IFI: Incremental fit index
