# Supplementary material for: Enhancing the work engagement of frontline nurses during the COVID-19 pandemic: the mediating role of affective commitment and perceived organizational support
Source: BMC Nurs. 2023 Dec 1;22:451. doi: 10.1186/s12912-023-01623-z (PMC10691007; doi:10.1186/s12912-023-01623-z)
Supplement: Supplementary file 1 — Supplementary Material 1 [file 12912_2023_1623_MOESM1_ESM.docx]

| **Appendix Table 1. Model-fitting Standards and the Fitting Index of the Final Model (n=842)** | | | | | | | | | | | |
| --- | --- | --- | --- | --- | --- | --- | --- | --- | --- | --- | --- |
| Model | Description | *χ^2^* | *df* | *p* | RMSEA | GFI | AGFI | TLI | NFI | IFI | CFI |
| Full Model | Full model | 3.632 | 2 | .162 | .031 | .998 | .987 | .994 | .997 | .999 | .999 |
| Model 1 | 3 factors: anxiety, PW, JE | 955.203 | 1 | .001 | .033 | .999 | .998 | .998 | .999 | .999 | .999 |
| Model 2 | 4 factors: anxiety, PW, AC, JE | 22.474 | 2 | .001 | .011 | .987 | .935 | .942 | .979 | .981 | .981 |
| Model 3 | 4 factors: anxiety, PW, POS, JE | .194 | 1 | .66 | .001 | .999 | .999 | .999 | .999 | .999 | .999 |

Note: AC, affective commitment; JE, job engagement; POS, perceived organizational support; PW, perceived workload.

| **Appendix Table 2. Estimates of the Standardized Regression Weights of the Models (n=842)** | | | | |
| --- | --- | --- | --- | --- |
| Significant path | Full Model | Model 1 | Model 2 | Model 3 |
| Anxiety --> Organizational support | -.252*** |  |  | -.252*** |
| Anxiety --> Perceived workload | .327*** | .327*** | .327*** | .327*** |
| Organizational support --> Affective commitment | .445*** |  |  |  |
| Anxiety --> Affective commitment | -.349*** |  | -.463*** |  |
| Perceived workload --> Affective commitment | .110*** |  | .117*** |  |
| Affective commitment --> Work engagement | .657*** |  | .773*** |  |
| Organizational support --> Work engagement | .136*** |  |  | .429*** |
| Anxiety --> Work engagement |  | -.456*** |  |  |
| Perceived workload --> Work engagement |  | .120*** |  | .114*** |

Note: ***p<.001 (two-tailed); Model 1: anxiety, perceived workload, job engagement; Model 2: anxiety, perceived workload, affective commitment, job engagement; Model 3: anxiety, perceived workload, perceived organizational support, job engagement.
